# Shaped Laser Pulses for Microsecond Time-Resolved Cryo-EM: Outrunning Crystallization during Flash Melting

**DOI:** 10.1021/acs.jpclett.4c00315

**Published:** 2024-04-11

**Authors:** Constantin R. Krüger, Nathan J. Mowry, Marcel Drabbels, Ulrich J. Lorenz

**Affiliations:** Laboratory of Molecular Nanodynamics, Ecole Polytechnique Fédérale de Lausanne (EPFL), CH-1015 Lausanne, Switzerland

## Abstract

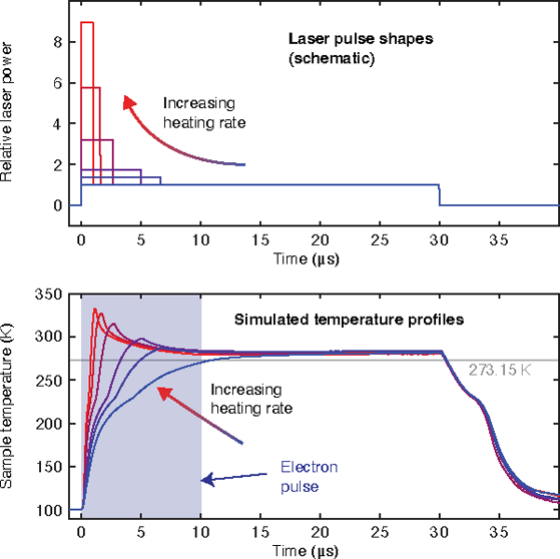

Water vitrifies if cooled at rates above 3 × 10^5^ K/s. In contrast, when the resulting amorphous ice is flash heated, crystallization occurs even at a more than 10 times higher heating rate, as we have recently shown. This may present an issue for microsecond time-resolved cryo-electron microscopy experiments, in which vitreous ice samples are briefly melted with a laser pulse because transient crystallization could potentially alter the dynamics of the embedded proteins. Here, we demonstrate how shaped microsecond laser pulses can be used to increase the heating rate and outrun crystallization. Time-resolved electron diffraction experiments reveal that the critical heating rate for amorphous solid water (ASW) is about 10^8^ K/s. Our experiments add to the toolbox of the emerging field of microsecond time-resolved cryo-electron microscopy by demonstrating a straightforward approach for avoiding crystallization during laser melting and for achieving significantly higher heating rates, which paves the way for nanosecond time-resolved experiments.

If water is cooled at a rate of over 3 × 10^5^ K/s,^1^ it vitrifies and forms hyperquenched glassy water (HGW), a type of amorphous ice, once the glass transition temperature of 136 K is reached.^2^ The successful vitrification of aqueous samples has laid the foundation for cryo-electron microscopy (cryo-EM),^3^ which is on its way to become the preferred tool of structural biologists.^4^ By outrunning crystallization during the vitrification process, the structure of proteins can be preserved in a frozen-hydrated state. This makes it possible to image them with an electron microscope and use single-particle reconstruction techniques to obtain their three-dimensional structures.^3^

We recently demonstrated that the vitrification process cannot simply be reversed by heating amorphous ice samples at a similar rate. Instead, partial crystallization occurs even at a heating rate of over 5 × 10^6^ K/s.^5^ This is a result of the different temperature dependence of the nucleation and growth rates of supercooled water.^2,6^ During flash heating, an amorphous ice sample first traverses a temperature range in which fast nucleation occurs before it reaches higher temperatures, at which nucleation largely ceases but the growth rate surges so that the sample crystallizes rapidly. In contrast, during hyperquenching, the sequence of events is reversed, which slows the crystallization. The critical heating rate at which crystallization can be outrun during flash melting of an amorphous ice sample remains unknown.^5^

The partial crystallization of vitreous ice samples during flash melting^5,8^ also has important implications for microsecond time-resolved cryo-EM experiments, in which a cryo sample is flash melted with a microsecond laser pulse in order to allow protein dynamics to briefly occur while the sample is liquid.^7−9^ As the dynamics unfold, the heating laser is switched off, and the sample cools within microseconds and revitrifies, arresting the proteins in their transient configurations, which are subsequently imaged with single particle cryo-EM techniques. Near-atomic resolution reconstructions from revitrified cryo samples demonstrate that the transient crystallization of the sample during laser melting does not alter the structure of embedded particles.^10,11^ This is consistent with the observation that high-resolution reconstructions can be obtained from fully devitrified cryo samples.^12^ However, it is conceivable that transient crystallization may affect the structure of more fragile proteins and loosely bound complexes or alter their dynamics. It is therefore desirable to avoid crystallization altogether. Here, we show that this can be achieved by using shaped laser pulses with an intense leading edge, which makes it possible to dramatically increase the heating rate. Moreover, by systematically varying the heating rate and using time-resolved electron diffraction to probe for crystallization during the melting process, we can determine the critical heating rate.

Experiments are performed with a time-resolved transmission electron microscope that we have previously described (Supplementary Methods 1).^13,14^ As illustrated in Figure 1a, a few-layer graphene sheet, supported by a holey gold film (2 μm holes) on a 600 mesh gold grid, serves as the sample support, which is held at a temperature of 100 K, and water vapor is deposited *in situ* to grow a 100 nm thick layer of ASW, an amorphous ice that is structurally similar to HGW.^5^ We then flash melt the sample in the center of a grid square with a temporally shaped 30 μs laser pulse (532 nm) and probe whether crystallization occurs by recording a diffraction pattern during the first 10 μs of the melting process with an intense, high-brightness electron pulse^13,14^ (Figure 1b).

**Figure 1:**
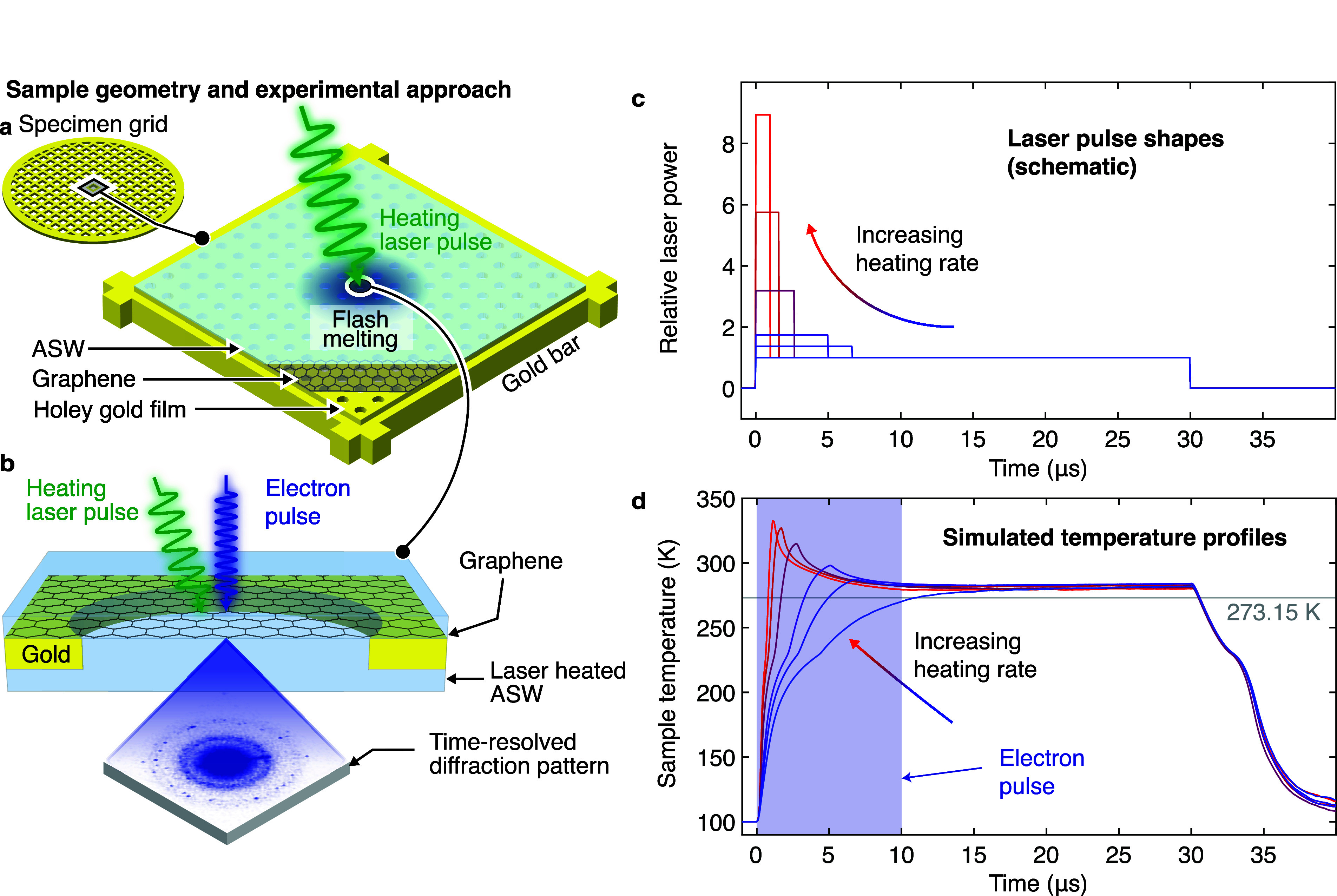
Illustration of the experimental approach and simulation of the temperature evolution of the sample. (a) Illustration of the sample geometry. A gold mesh supports a holey gold film covered with multilayer graphene, onto which we deposit a 100 nm thick layer of ASW (100 K sample temperature). We then use a shaped microsecond laser pulse to locally melt the sample. (b) We probe for crystallization during the melting process by capturing a diffraction pattern with an intense, 10 μs electron pulse (200 kV accelerating voltage). (c) Schematic illustration of the laser pulse shapes. The heating rate is varied by changing the intensity and duration of the initial spike while keeping its integral constant. (d) Simulation of the temperature evolution of the sample under irradiation with the shaped laser pulses illustrated in (c). The simulation uses the experimentally determined pulse shapes shown in Supplementary Methods 2. The electron pulse probes the first 10 μs of the melting process.

Figure 1c schematically illustrates representative shapes of the heating laser pulses, with simulations of the corresponding temperature evolution of the sample shown in Figure 1d (Supplementary Methods 2). With a simple rectangular pulse (blue), the sample heats within ∼11 μs, before its temperature stabilizes at about 280 K, as previously determined.^5^ Once the laser is switched off, the sample cools within a few microseconds and vitrifies.^7^ We increase the heating rate during the melting process by adding an initial spike to the laser pulse (red and purple curves). By changing the intensity and duration of this spike while keeping its integral approximately constant, we can adjust the heating rate in the range between 1.6 × 10^7^ and 3.0 × 10^8^ K/s, with the lowest heating rate corresponding to the simple rectangular laser pulse and the highest rate to a 450 ns spike of more than 17 times the laser power (Supplementary Methods 2). The heating rates we report correspond to averages of the simulated rates between 100 and 273 K.

Figure 2a shows typical diffraction patterns recorded during the first 10 μs of the flash melting process with a single electron pulse for heating rates between 1.6 × 10^7^ and 2.2 × 10^8^ K/s. At low heating rates, distinct diffraction spots are visible on top of the broad background of the water diffraction pattern, indicating that the sample has partially crystallized, whereas at higher heating rates, these diffraction features become increasingly fainter. Figure 2b shows the corresponding azimuthally averaged diffraction patterns from the sum of the five or six experiments. These diffraction patterns represent weighted averages of the different temperatures that the sample has explored. At low heating rates, the patterns resemble that of amorphous ice, with a large spacing between the first two diffraction maxima, and exhibit a small contribution from stacking disordered ice. At high heating rates, the sample spends more time at higher temperatures, so that the diffraction pattern increasingly resembles that of stable water. This causes the diffraction intensity to drop and the first diffraction maximum to shift to higher momentum transfer, while the second maximum slightly moves in the opposite direction.^15^

**Figure 2:**
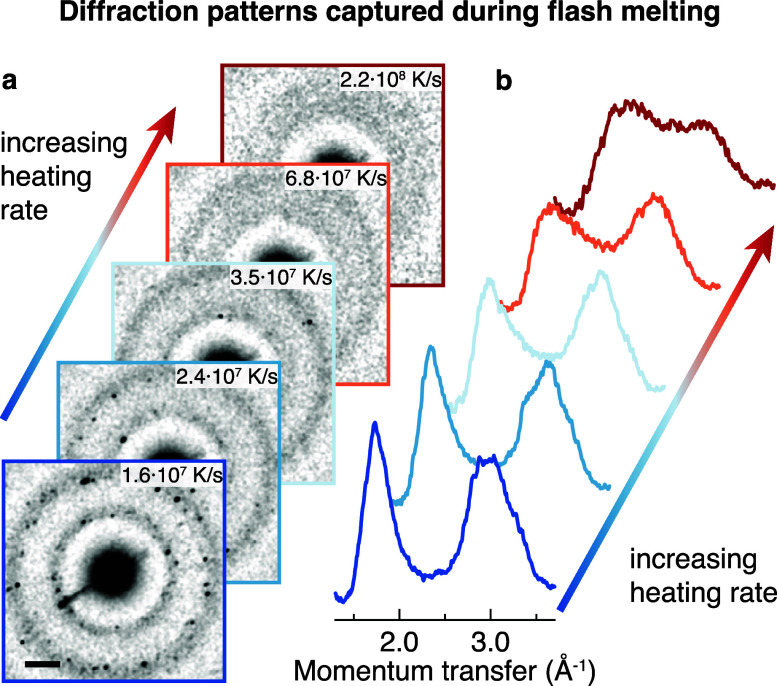
Diffraction patterns captured during flash melting. (a) Examples of diffraction patterns (Gaussian filtered) recorded during the first 10 μs of the melting process with a single electron pulse for heating rates between 1.6 × 10^7^ and 2.2 × 10^8^ K/s. Scale bar: 1 Å^–1^. (b) Azimuthally averaged diffraction patterns for the heating rates in a from the sum of five experiments.

We obtain the critical heating rate by determining the intensity of crystalline features in the diffraction patterns recorded during flash melting (Figure 2b) as a function of the heating rate. To this end, we decompose the diffraction patterns into the three components shown in Figure 3a. The diffraction patterns of HGW (green) and liquid water at ∼280 K (purple) serve to capture low- and high-temperature structures of liquid water, respectively, while the diffraction pattern of stacking disordered ice (black) is used to describe the crystalline fraction of the sample (Supplementary Methods 3). As shown in Figure 3b, the experimental diffraction patterns (dashed lines) can be reasonably well described by such weighted sums (solid lines). Figure 3c displays the weights of the three components as a function of the heating rate. As expected, the contribution of the high-temperature structure (purple) increases with heating rate, while the contribution of the low-temperature structure (green) decreases, approaching zero at the highest heating rate. The crystalline component (black, detail in Figure 3d) has a weight of about 0.11 at the lowest heating rate. This is roughly consistent with our previous estimate that about a third of the sample crystallizes during flash melting with a rectangular laser pulse, if one takes into account that the 10 μs electron pulse probes the sample during the course of the crystallization process. As the heating rate increases, the weight of the crystalline component drops rapidly and approaches zero at a heating rate of about 1 × 10^8^ K/s, which we therefore identify as the critical heating rate.

**Figure 3:**
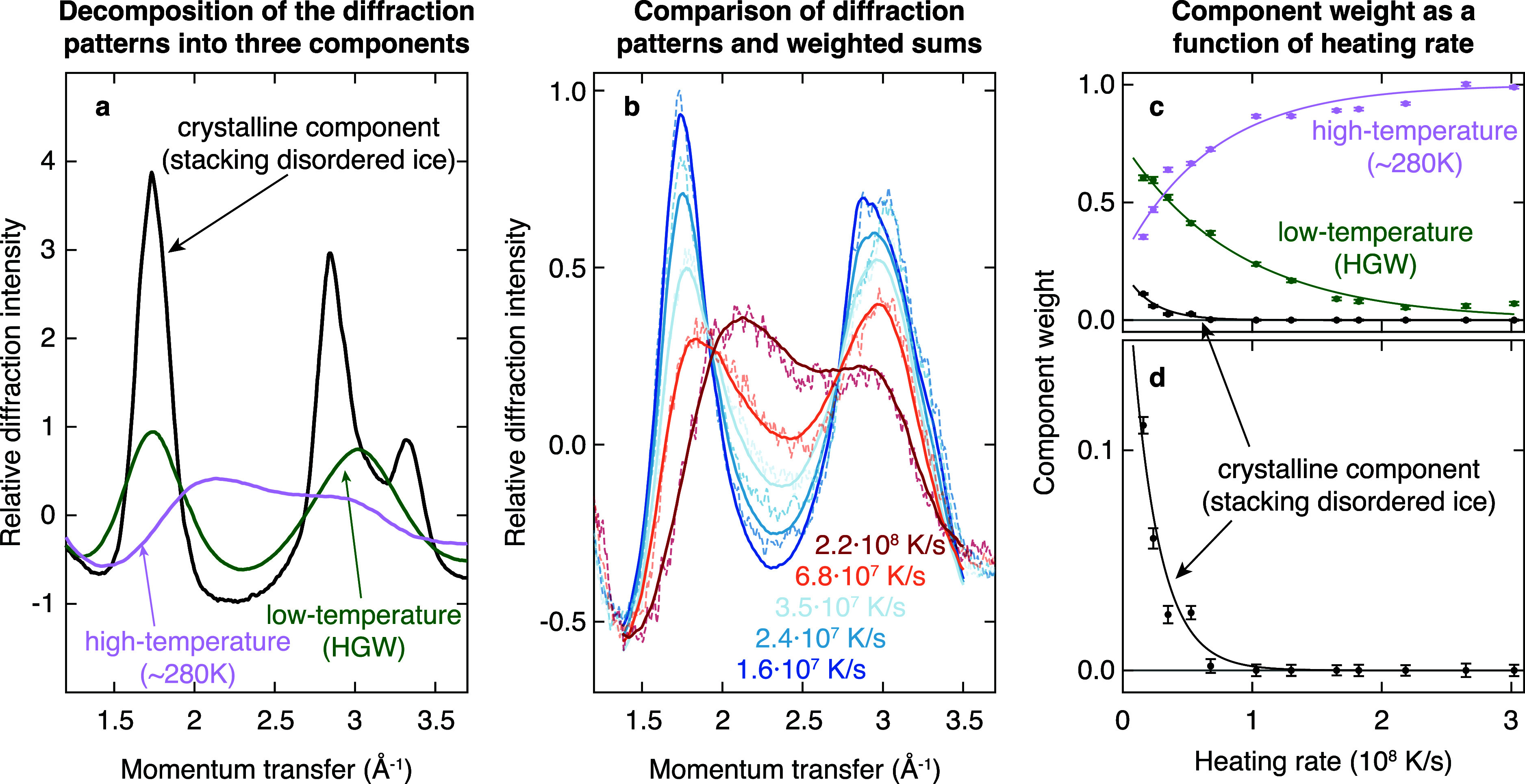
Determination of the critical heating rate for outrunning crystallization. (a) The diffraction patterns recorded during the melting process (Figure 2b) can be well reproduced by a weighted sum of the diffraction patterns of a low-temperature component (green, HGW at 100 K), a high-temperature component (purple, water at ∼280 K), and a crystalline component (black, stacking disordered ice at 100 K). (b) Weighted sums of the components in a (solid lines) show good agreement with the experimental diffraction patterns (dashed lines). (c, d) Weight of the three components in a as a function of heating rate. The weight of the crystalline component approaches zero at heating rates exceeding 10^8^ K/s, marking the critical heating rate. Error bars represent standard errors of the fit. The solid lines provide a guide to the eye and are derived from exponential fits.

In conclusion, we have determined that during flash melting of ASW samples, the critical heating rate for outrunning crystallization is 10^8^ K/s, more than 2 orders of magnitude higher than the critical cooling rate during vitrification of about 3 × 10^5^ K/s.^1^ Our previous experiments have shown that HGW samples crystallize more rapidly during flash melting than ASW samples, consistent with a 5 times higher nucleation rate.^5^ This allows us to estimate that HGW samples should have a 1.5 times higher critical heating rate (Supplementary Methods 4). Note that surface nucleation likely dominates the crystallization process in our thin film samples.^5,16^ Therefore, the critical heating rate obtained here represents an upper limit for bulk samples. We similarly expect a lower critical heating rate for typical cryo samples used in microsecond time-resolved cryo-EM experiments because such samples are usually buffered, and crystallization is known to slow with increasing salt concentration.^1^

Our experiments demonstrate that shaped laser pulses with an intense leading edge provide a straightforward approach for achieving faster heating rates in time-resolved cryo-EM experiments and for avoiding crystallization during flash melting as well as its potentially deleterious effects on particle structure and dynamics. We find that while typical cryo samples transiently crystallize during flash melting with a rectangular laser pulse, crystallization can be comfortably outrun with approximately a 1 μs initial spike of about 9 times the laser power (Supplementary Methods 5). Unless desired, excessive heating of the sample should be avoided by carefully choosing the intensity of the spike based on the heat transfer properties of the sample, which can be characterized experimentally.^15^

Shaped laser pulses with an intense leading edge can also be used to improve the time resolution of our technique. As we have recently demonstrated, a straightforward strategy for initiating protein dynamics consists in releasing a caged compound^17^ while the sample is still in its frozen state.^9^ Because the matrix of vitreous ice prevents the embedded proteins from undergoing dynamics, the liberated compound then becomes active only once the sample is liquid. In such experiments, the time resolution is determined both by the heating rate, which determines how fast the sample can be melted and dynamics can be initiated, and by the cooling rate, which dictates how fast the sample can be revitrified and protein motions can be arrested. With a rectangular heating laser pulse, several microseconds can elapse before the sample temperature has stabilized, depending on the heat transfer properties of the sample.^5,7,15^ Moreover, if partial crystallization occurs during flash melting, several microseconds more may be required to melt the crystallites that have formed.^5^ Particles will therefore be released from their crystalline matrix and start undergoing dynamics at different times, which further lowers the time resolution. As we have shown here, this can be avoided by using shaped laser pulses, which can outrun crystallization and reduce the melting time of the sample to below 600 ns. With suitable hardware, an even shorter melting time can be achieved so that its contribution to the time resolution becomes negligible. Together with sample geometries that allow for faster heat dissipation and thus shorter cooling times, this paves the way for nanosecond time-resolved cryo-EM experiments.

## Data Availability

All data are available at https://doi.org/10.5281/zenodo.10640850.

## Supporting Information Available

Additional experimental methods, lase pulse shape characterization, decomposition of diffraction patterns, estimation of critical heating rate of HGW and flash melting of cryo sample (PDF)
Transparent Peer Review report available (PDF)
