# Supplementary material for: Shaped Laser Pulses for Microsecond Time-Resolved Cryo-EM: Outrunning Crystallization during Flash Melting
Source: J Phys Chem Lett. 2024 Apr 11;15(16):4244–8. doi: 10.1021/acs.jpclett.4c00315 (PMC11057027; doi:10.1021/acs.jpclett.4c00315)
Supplement: Supplementary file 1 — jz4c00315_si_001.pdf [file jz4c00315_si_001.pdf]

Supplementary Information

Shaped Laser Pulses for Microsecond Time-Resolved Cryo-EM: Outrunning Crystallization During Flash Melting

Constantin R. Krüger†, Nathan J. Mowry†, Marcel Drabbels, and Ulrich J. Lorenz*

Affiliation: Ecole Polytechnique Fédérale de Lausanne (EPFL), Laboratory of Molecular Nanodynamics, CH-1015 Lausanne, Switzerland

This PDF file includes:

1 Experimental methods

2 Characterization of the laser pulse shapes and determination of the heating rates

3 Decomposition of the diffraction patterns recorded during flash melting into three components

4 Estimate of the critical heating rate for HGW samples

5 Flash melting of a typical cryo sample

† These authors contributed equally.

* To whom correspondence should be addressed. E-mail: ulrich.lorenz@epfl.ch

1 Experimental methods

Experiments are performed with a JEOL 2010F transmission electron microscope that we have modified for time-resolved experiments.[1,2] Sample supports are prepared by transferring 6-8 layer graphene onto Quantifoil (Au) R2/1 (N1-A15nAu60-50) specimen grids (50 nm thick holey gold film with 2 μm diameter holes, 1 μm apart on 600 mesh gold).[3,4] The sample is held at a temperature of 100 K, and a 100 nm thick layer of ASW is deposited *in situ* as previously described.[3,4] Here, the cold shield surrounding the sample is cooled to liquid nitrogen temperature in order to allow for faster deposition rates. The time-resolved electron diffraction experiments follow the same sequence of events as before.[3,4] Static diffraction patterns of the sample are captured before and after each experiment, and a time-resolved diffraction pattern is captured during the first 10 μs of the laser melting process to probe for crystallization. To this end, we use an intense, high-brightness electron pulse, which is obtained by temporarily laser heating the tip of our Schottky emitter to extreme temperatures and thus increasing emission to near its limit. This boosted electron beam is then chopped into a 10 μs pulse with an electrostatic deflector.[1,2] After the experiment, the sample is evaporated, and a fresh ASW sample is deposited. The diffraction patterns are analyzed as previously described and are shown with the diffraction background subtracted.[3,4]

2 Characterization of the laser pulse shapes and determination of the heating rates

In order to systematically vary the heating rate, we change the amplitude and duration of the initial spike of the shaped laser pulse, while keeping the integral of the spike approximately constant. Figure S1 shows the experimental laser pulse shapes, as recorded with a photodiode (averages of 10). The area under the initial spike varies by less than 10 % across the different pulse shapes.

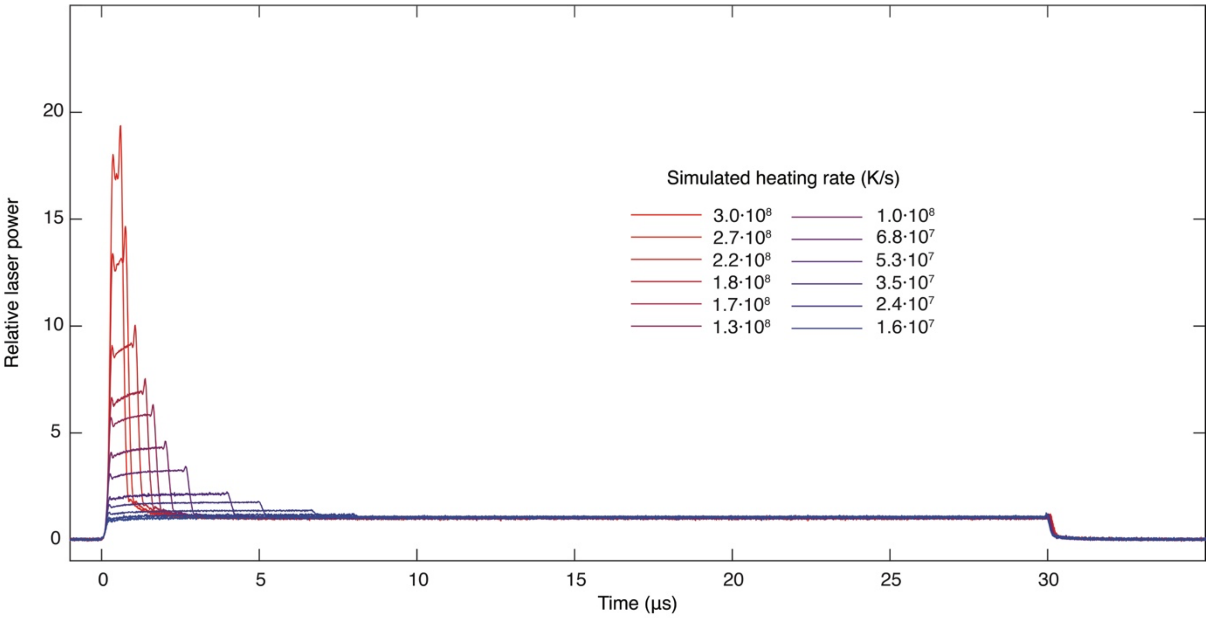

Figure S1. Experimental laser pulse shapes as determined with a photodiode. The simulated heating rates are listed for each pulse shape.

The amplitude and duration of the spike are listed in Table 1 for each laser pulse shape, together with the heating rate, as determined from heat transfer simulations. The temperature evolution of the sample is simulated with COMSOL Multiphysics 6.1, with the simulation parameters as previously described.[3,4] We simulate the experimentally determined laser pulse shape and adjust the simulated laser power such that at the end of a rectangular laser pulse, the sample temperature plateaus at 280 K, as previously determined in a similar experiment.[4] Within the estimated error of this plateau temperature of less than ~10 K, the heating rate changes only marginally. We report the average heating rate of the sample between 100 K and 273 K in the volume probed by the electron pulse (~1.5 µm beam diameter). The largest temperature difference across this volume occurs at the highest heating rate and briefly reaches about 50 K.

Relative spike amplitude	Spike duration (µs)	Simulated heating rate (K/s)
17.1	0.45	$3.0 \cdot 10^8$
13.0	0.64	$2.7 \cdot 10^8$
8.94	0.97	$2.2 \cdot 10^8$
6.74	1.30	$1.8 \cdot 10^8$
5.75	1.59	$1.7 \cdot 10^8$
4.20	2.00	$1.3 \cdot 10^8$
3.19	2.69	$1.0 \cdot 10^8$
2.14	4.02	$6.8 \cdot 10^7$
1.74	5.09	$5.3 \cdot 10^7$
1.37	6.68	$3.5 \cdot 10^7$
1.16	7.99	$2.4 \cdot 10^7$
1.00	—	$1.6 \cdot 10^7$

Table 1. Parameters of the shaped laser pulses and simulated heating rates. The amplitude of the initial spike is indicated in multiples of the laser power of a simple rectangular pulse. Both the amplitude and duration are measured with a photodiode.

3 Decomposition of the diffraction patterns recorded during flash melting into three components

In order to determine whether crystallization occurred during flash melting, we decompose the diffraction patterns captured during the first 10 µs of the melting process into a high- and a low-temperature component as well as a crystalline component. The weights of the components are determined with least-squares minimization.

For the low-temperature component, we use the diffraction pattern of the HGW sample that is obtained after each laser pulse, where we have averaged diffraction patterns from 73 individual experiments, each recorded with 20 boosted electron pulses of 10 µs duration, fired at 10 Hz repetition rate.

For the high-temperature component, we use the diffraction pattern of liquid water (~280 K), which we record at the end of the laser pulse with a 10 μs boosted electron pulse. The diffraction pattern in Fig. 3a is an average of 4 patterns, smoothed with a rolling average filter.

For the crystalline component, we use the diffraction pattern of a devitrified sample, which predominantly consists of stacking disordered ice (20 boosted electron pulses of 10 μs duration, fired at 10 Hz repetition rate). The sample was prepared by irradiating a freshly deposited ASW sample with 10 laser pulses of 30 μs duration, with the laser power reduced to two thirds of the power needed for achieving melting and revitrification. At this laser power, the sample temperature plateaus in the deeply supercooled regime, and devitrification occurs.

4 Estimate of the critical heating rate for HGW samples

We have previously shown that HGW samples exhibit faster crystallization kinetics during flash melting than ASW samples, a difference that can be explained if one assumes that the nucleation rate of HGW is about 5 times as large as that of ASW.[4] This allows us to estimate the critical heating rate for HGW samples with the help of simulations of the crystallization kinetics. Our model, which we have detailed previously,[4] is based on classical nucleation and growth theory[5,6] and uses experimental nucleation and growth rates.[7–12] In the deeply supercooled regime, where the nucleation rate goes through a maximum, experimental values are largely not available.[13] Moreover, in our thin film samples, surface nucleation likely plays an important role, which increases the nucleation rate.[4] We therefore treat the maximum nucleation rate of deeply supercooled water as a free parameter.

We begin by simulating the flash melting of an ASW sample with a constant heating rate of $1\cdot10^8$ K/s, which corresponds to the critical heating rate that we have determined experimentally. We then adjust the maximum nucleation rate of deeply supercooled water such that only a small fraction of the sample of 0.1 % has crystallized when the sample reaches the melting point. In order to simulate the flash melting of HGW, we then increase the maximum nucleation rate by a factor of 5, which increases the crystalline fraction of the sample when it reaches the melting point to 0.5 %. We find that in order to maintain the crystalline fraction at 0.1 %, the heating rate has to be increased by a factor of 1.5. This allows us to estimate that the critical heating rate for HGW samples is 1.5 times higher than for ASW.

Note that this estimate is not sensitive to our choice of the crystalline fraction that is observed at the critical heating and barely changes if we reduce the crystalline fraction by one or two orders of magnitude.

5 Flash melting of a typical cryo sample

While typical cryo samples transiently crystallize during flash melting with a rectangular laser pulse, crystallization can be outrun with a shaped laser pulse is used. This is shown in Fig. S2 for a cryo sample of the 50S subunit of the ribosome (20 mM HEPES at pH 7.5, 100mM NaCl, 2mM $MgCl_2$) on an UltrAuFoil R1.2/1.3 (N1 - A14nAu30 – 01) specimen grid (50 nm thick holey gold film with 1.2 µm diameter holes, 1.3 µm apart on 300 mesh gold). As shown in Fig. S2a, when the sample is flash melted with a rectangular 30 µs laser pulse, transient crystallization is evident in a diffraction pattern recorded during the melting process (captured with a 10 µs electron pulse between 3 µs and 13 µs). In contrast, crystallization can be outrun with an initial spike of 0.94 µs duration and 8.94 times the laser power (Fig. S2b, diffraction pattern recorded between 0 µs and 10 µs).

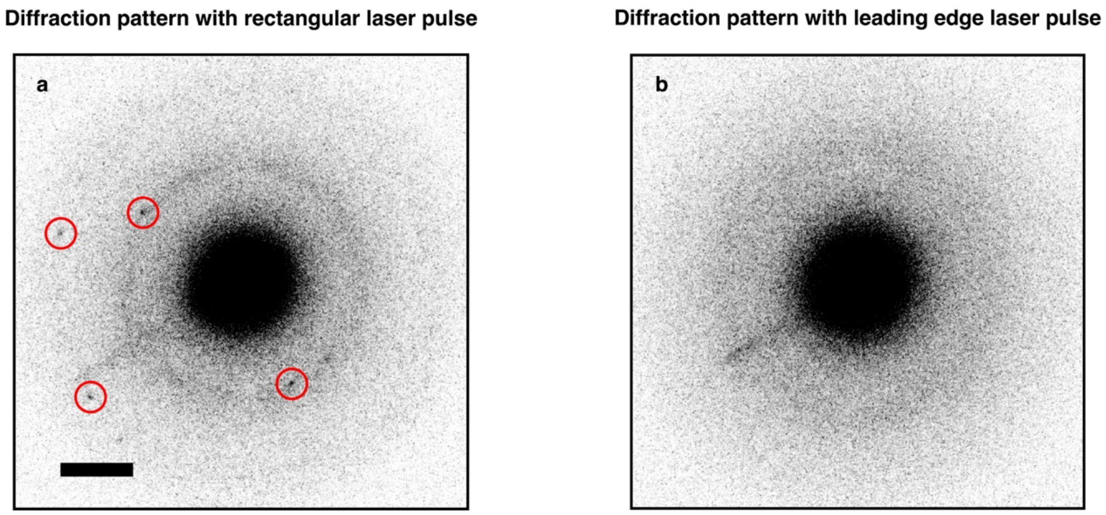

Figure S2. Diffraction patterns of a typical cryo sample recorded during flash melting with a rectangular and a shaped laser pulse. a Transient crystallization is evident during flash melting with a rectangular 30 µs laser pulse (crystalline diffraction features highlighted with circles). The time-resolved diffraction pattern was captured with a 10 µs electron pulse between 3 µs and 13 µs.

b Crystallization can be outrun with a shaped laser pulse (0.94 μs initial spike with 8.94 times the laser power). The time-resolved diffraction pattern was recorded between 0 μs and 10 μs. Scale bar, 1 $Å^{-1}$.

References

(1) Bongiovanni, G.; Olshin, P. K.; Drabbels, M.; Lorenz, U. J. Intense Microsecond Electron Pulses from a Schottky Emitter. *Appl. Phys. Lett.* **2020**, *116* (23), 234103.

(2) Olshin, P. K.; Bongiovanni, G.; Drabbels, M.; Lorenz, U. J. Atomic-Resolution Imaging of Fast Nanoscale Dynamics with Bright Microsecond Electron Pulses. *Nano Lett.* **2021**, *21* (1), 612–618.

(3) Krüger, C. R.; Mowry, N. J.; Bongiovanni, G.; Drabbels, M.; Lorenz, U. J. Electron Diffraction of Deeply Supercooled Water in No Man's Land. *Nat. Commun.* **2023**, *14* (1), 2812.

(4) Mowry, N. J.; Krüger, C. R.; Bongiovanni, G.; Drabbels, M.; Lorenz, U. J. Flash Melting Amorphous Ice. *ArXiv Prepr. ArXiv231211579* **2023**.

(5) Debenedetti, P. G. Metastable Liquids: Concepts and Principles. *Princeton University Press: Princeton.* **1997.**

(6) Backus, E. H. G.; Grecea, M. L.; Kleyn, A. W.; Bonn, M. Surface Crystallization of Amorphous Solid Water. *Phys. Rev. Lett.* **2004**, *92* (23), 236101.

(7) Safarik, D. J.; Mullins, C. B. The Nucleation Rate of Crystalline Ice in Amorphous Solid Water. *J. Chem. Phys.* **2004**, *121* (12), 6003–6010.

(8) Laksmono, H.; McQueen, T. A.; Sellberg, J. A.; Loh, N. D.; Huang, C.; Schlesinger, D.; Sierra, R. G.; Hampton, C. Y.; Nordlund, D.; Beye, M.; Martin, A. V.; Barty, A.; Seibert, M. M.; Messerschmidt, M.; Williams, G. J.; Boutet, S.; Amann-Winkel, K.; Loerting, T.; Pettersson, L. G. M.; Bogan, M. J.; Nilsson, A. Anomalous Behavior of the Homogeneous Ice Nucleation Rate in "No-Man's Land." *J. Phys. Chem. Lett.* **2015**, *6* (14), 2826–2832.

(9) Murray, B. J.; Broadley, S. L.; Wilson, T. W.; Bull, S. J.; Wills, R. H.; Christenson, H. K.; Murray, E. J. Kinetics of the Homogeneous Freezing of Water. *Phys. Chem. Chem. Phys.* **2010**, *12* (35), 10380.

(10) Stöckel, P.; Weidinger, I. M.; Baumgärtel, H.; Leisner, T. Rates of Homogeneous Ice Nucleation in Levitated H2O and D2O Droplets. *J. Phys. Chem. A* **2005**, *109* (11), 2540–2546.

(11) Riechers, B.; Wittbracht, F.; Hütten, A.; Koop, T. The Homogeneous Ice Nucleation Rate of Water Droplets Produced in a Microfluidic Device and the Role of Temperature Uncertainty. *Phys. Chem. Chem. Phys.* **2013**, *15* (16), 5873.

(12) Stan, C. A.; Schneider, G. F.; Shevkoplyas, S. S.; Hashimoto, M.; Ibanescu, M.; Wiley, B. J.; Whitesides, G. M. A Microfluidic Apparatus for the Study of Ice Nucleation in Supercooled Water Drops. *Lab. Chip* **2009**, *9* (16), 2293.

(13) Gallo, P.; Amann-Winkel, K.; Angell, C. A.; Anisimov, M. A.; Caupin, F.; Chakravarty, C.; Lascaris, E.; Loerting, T.; Panagiotopoulos, A. Z.; Russo, J.; Sellberg, J. A.; Stanley, H. E.; Tanaka, H.; Vega, C.; Xu, L.; Pettersson, L. G. M. Water: A Tale of Two Liquids. *Chem. Rev.* **2016**, *116* (13), 7463–7500.
