# Supplementary material for: Shaped Laser Pulses for Microsecond Time-Resolved Cryo-EM: Outrunning Crystallization during Flash Melting
Source: J Phys Chem Lett. 2024 Apr 11;15(16):4244–8. doi: 10.1021/acs.jpclett.4c00315 (PMC11057027; doi:10.1021/acs.jpclett.4c00315)
Supplement: Supplementary file 2 — jz4c00315_si_002.pdf [file jz4c00315_si_002.pdf]

jz-2024-00315e.R1

Name: Peer Review Information for "Shaped Laser Pulses for Microsecond Time-Resolved Cryo-EM: Outrunning Crystallization During Flash Melting"

First of Reviewer Comments

Reviewer: 1

Comments to the Author

This is a superbly written paper presenting exciting and important results. Publish asap as is!

Reviewer: 2

Comments to the Author

The manuscript by Kruger and co-workers describes the optically triggered crystallization dynamics of amorphous solid water thin films at high heating rates exceeding 3x108 K/s. Before the water film melts by the incident optical pulses, transient crystallization may occur depending on the heating rate which is modified by the temporal shape of the optical trigger pulse. The crystallization of in-situ prepared ice films is recorded by transmission electron microscopy with pulsed electron beams.

The experiments reported in the manuscript are to some extend connected to a recent publication by the Lorenz group (Ref. 15 of the manuscript) in which the transient electron diffraction pattern of amorphous ice at intermediate temperature was reported. In addition, in a current arXiv paper (Ref. 5) the Lorenz group reports the timescale of crystallization dynamics starting from two different amorphous ice forms and utilizing optical pulses with a rectangular temporal intensity profile.

Different from these publications, the current manuscript places the focus of the investigation on the heating-rate dependence of the crystallization, and it is demonstrated that at heating rates above about 108 K/s (achieved by an intensity spike early in the optical pulse) amorphous ice directly transforms into the liquid state without transient crystallization. This fact is interesting since the rate is much higher than the required cooling rate to obtain amorphous ice. From an applied point of view the finding is important, since the optically triggered transient melting of amorphous ice with embedded proteins was recently demonstrated by the Lorenz group to offer a viable approach for time-resolved cryo-electron microscopy with microsecond temporal resolution. Despite the related results already published results by the authors, I believe the

manuscript could be suitable for the broader readership of Journal of Physical Chemistry Letter, in particular, due to the importance for future developments in cryo-electron microscopy. However, in my opinion the manuscript requires improvement in its accessibility for a broader non-specialist audience considering the specific questions and comments listed below:

(1) The terms “hyperquenched glassy water (HGW)”, i.e. water subjected to a high cooling rate , and “amorphous solid water (ASW)” obtained by water deposition at low temperatures should be explicitly explained in the manuscript.

(2) The authors state that nucleation for forming crystalline ice is likely occurring at the surface. Are there any indications (for example using electron tomography) if crystals predominantly form at the water/vacuum or the water/graphene interface?

(3) The authors mention that the transient diffraction patterns are recorded with intense, high-brightness electron pulses. Is there a quantitative characterization of the pulse brightness? It would be helpful to provide more details on the TEM imaging conditions and the pulse generation methodology. I also recommend detailing what is meant by “boosted” electron pulses (cf. Supplement).

(4) Are the diffraction patterns shown in Fig. 2 obtained with a single electron pulse or are these averaged over many experiments?

(5) As the authors correctly stated, for embedding proteins in HGW, solutions are typically buffered so that transient crystallization during rapid laser-heating is less likely. Considering this statement, how is the heat-rate dependence investigation reported in the manuscript important for time-resolved cryo-electron microscope? Similarly, in the conclusion, the possibility of nanosecond time-resolved cryo-electron microscopy is mentioned and linked to fast heating rates achieved by optical pulse shaping. Are heating rates currently the limiting factor in cryo-electron microscopy?

Author's Response to Peer Review Comments:

Dear Editor,

We are submitting a revised version of the manuscript and SI that addresses the reviewers’ comments together with a point-by-point response.

We hope to receive your decision soon.

Best regards,

Ulrich J. Lorenz

We would like to thank the reviewers for their detailed reviews and are attaching our responses below.

Reviewer #1

Recommendation: This paper represents a significant new contribution and should be published as is.

Comments:

This is a superbly written paper presenting exciting and important results. Publish asap as is!

Additional Questions:

Urgency: Top 10%

Significance: Top 10%

Novelty: Top 10%

Scholarly Presentation: Top 10%

Is the paper likely to interest a substantial number of physical chemists, not just specialists working in the authors' area of research?: Yes

Reviewer #2

Recommendation: This paper is probably publishable, but major revision is needed; I do not need to see future revisions.

Comments:

The manuscript by Kruger and co-workers describes the optically triggered crystallization dynamics of amorphous solid water thin films at high heating rates exceeding 3x108 K/s. Before the water film melts by the incident optical pulses, transient crystallization may occur depending on the heating rate which is modified by the temporal shape of the optical trigger pulse. The crystallization of in-situ prepared ice films is recorded by transmission electron microscopy with pulsed electron beams.

The experiments reported in the manuscript are to some extend connected to a recent publication by the Lorenz group (Ref. 15 of the manuscript) in which the transient electron diffraction pattern of amorphous ice at intermediate temperature was reported. In addition, in a current arXiv paper (Ref. 5) the Lorenz group reports the timescale of crystallization dynamics starting from two different amorphous ice forms and utilizing optical pulses with a rectangular temporal intensity profile.

Different from these publications, the current manuscript places the focus of the investigation on the heating-rate dependence of the crystallization, and it is demonstrated that at heating rates above about 108 K/s (achieved by an intensity spike early in the optical pulse) amorphous ice directly transforms into the liquid state without transient crystallization. This fact is interesting since the rate is much higher than the required cooling rate to obtain amorphous ice. From an applied point of view the finding is important, since the optically triggered transient melting of amorphous ice with embedded proteins was recently demonstrated by the Lorenz group to offer a viable approach for time-resolved cryo-electron microscopy with microsecond temporal resolution. Despite the related results already published results by the authors, I believe the manuscript could be suitable for the broader readership of Journal of Physical Chemistry Letter, in particular, due to the importance for future developments in cryo-electron microscopy. However, in my opinion the manuscript requires improvement in its accessibility for a broader non-specialist audience considering the specific questions and comments listed below:

(1) The terms “hyperquenched glassy water (HGW)”, i.e. water subjected to a high cooling rate , and “amorphous solid water (ASW)” obtained by water deposition at low temperatures should be explicitly explained in the manuscript.

We realize that while we explain in the introduction that HGW is formed through rapid cooling, we have not explained the nature of ASW. We have changed the description of the experimental procedure to clarify that ASW is obtained through the deposition of water vapor.

(2) The authors state that nucleation for forming crystalline ice is likely occurring at the surface. Are there any indications (for example using electron tomography) if crystals predominantly form at the water/vacuum or the water/graphene interface?

This statement is based on previous work, which has shown that surface nucleation dominates in thin film samples (Backus, E. H. G., Grecea, M. L., Kleyn, A. W. & Bonn, M. Surface Crystallization of Amorphous Solid Water. *Phys. Rev. Lett.* **92**, 236101 (2004).) We realize that Ref. 16 in our manuscript is in fact the wrong reference, which we have corrected.

(3) The authors mention that the transient diffraction patterns are recorded with intense, highbrightness electron pulses. Is there a quantitative characterization of the pulse brightness? It would be helpful to provide more details on the TEM imaging conditions and the pulse generation methodology. I also recommend detailing what is meant by "boosted" electron pulses (cf. Supplement).

We have detailed the generation and characterization of the electron pulses in Refs. 13, 14, as indicated in the main text. We have also clarified this point in the SI, in particular that "boosted" refers to the method of generation of the electron pulses, which involves heating the tip of our Schottky emitter to extreme temperatures with a laser beam in order to boost emission to near its limit.

(4) Are the diffraction patterns shown in Fig. 2 obtained with a single electron pulse or are these averaged over many experiments?

We have clarified that these patterns were recorded with a single electron pulse.

(5) As the authors correctly stated, for embedding proteins in HGW, solutions are typically buffered so that transient crystallization during rapid laser-heating is less likely. Considering this statement, how is the heat-rate dependence investigation reported in the manuscript important for time-resolved cryoelectron microscope?

As stated in the main text, we show in Supplementary Methods 5 that typical cryo samples do crystallize during flash melting with a rectangular laser pulse and that shaped pulses with an intense leading edge are required to outrun crystallization.

Similarly, in the conclusion, the possibility of nanosecond time-resolved cryo-electron microscopy is mentioned and linked to fast heating rates achieved by optical pulse shaping. Are heating rates currently the limiting factor in cryo-electron microscopy?

We have clarified that an increase in the cooling rate must also be implemented in order to improve the time resolution.

Additional Questions:

Urgency: High

Significance: Top 10%

Novelty: High

Scholarly Presentation: High

Is the paper likely to interest a substantial number of physical chemists, not just specialists working in the authors' area of research?: Yes
